# Spectrum of coronary anomalies and their categorical approach: rare case series

**DOI:** 10.1093/jscr/rjac310

**Published:** 2022-07-04

**Authors:** Krishnaprasad Bashyal, Bhagawan Koirala, Anil Bhattarai, Ravi Kumar Baral, Prabhat Khakural, Samir Shakya, Prashiddha Bikram Kadel

**Affiliations:** Department of Cardiac Surgery, Manmohan Cardiothoracic Vascular and Transplant Center, TUTH, Kathmandu, Nepal; Department of Cardiac Surgery, Manmohan Cardiothoracic Vascular and Transplant Center, TUTH, Kathmandu, Nepal; Department of Cardiac Surgery, Manmohan Cardiothoracic Vascular and Transplant Center, TUTH, Kathmandu, Nepal; Department of Cardiac Surgery, Manmohan Cardiothoracic Vascular and Transplant Center, TUTH, Kathmandu, Nepal; Department of Cardiac Surgery, Manmohan Cardiothoracic Vascular and Transplant Center, TUTH, Kathmandu, Nepal; Department of Pediatric Cardiology, Manmohan Cardiothoracic Vascular and Transplant Center, TUTH, Kathmandu, Nepal; Department of Cardiac Surgery, Manmohan Cardiothoracic Vascular and Transplant Center, TUTH, Kathmandu, Nepal

## Abstract

The incidence of coronary artery anomalies (CAAs) is 0.2–1.2% of the population. Its paradox of being a rare entity with presentation ranging from sudden cardiac death, congestive heart failure, myocardial infarction to being clinically silent, asserts a challenge to its treating physician. Among the various major categories of CAA, we describe four different types of these anomalies in our retrospective evaluation over 2 years. They include – coronary cameral fistula with coronary aneurysm, congenital atresia of left main, anomalous aortic origin of left anterior descending (LAD) and circumflex artery (LCx) with malignant LAD course, anomalous origin of left coronary artery from pulmonary artery (ALCAPA). Although the child with ALCAPA succumbed despite every possible and available timely efforts, other patients had good postoperative recovery and a brief hospital stay.

## INTRODUCTION

Coronary artery anomalies (CAAs) are an exceptionally rare entity and diagnosed in <1% of population [1, 2], but this incidence might be even lower in developing nations like Nepal where access to specialized healthcare in many areas is privileged. Angelini *et al*. [2] defined CAA as any coronary pattern with a feature (number of ostia, proximal course, termination, etc.) rarely encountered in general population.

Clinical relevance for these anomalies is that some of them maybe aberrations but not posing any threat to the myocardium, whereas certain conditions like anomalous origin of left coronary artery from pulmonary artery (ALCAPA) present with myocardial ischemia in early infancy and mandate prompt surgical intervention. Separate origin of left anterior descending (LAD) and circumflex artery (LCx) is reported as commonest CAA followed by origin of LCx from right coronary artery (RCA) with incidence of 0.41–0.37%, respectively [3]. In athletes under 35 years, CAA is the second most common cause of sudden cardiac death (SCD) after only hypertrophic cardiomyopathy [4, 5]. An anomalous origin of LAD from right sinus of valsalva (RSOV) with a malignant course traversing between the aorta and pulmonary artery (PA) is the most frequently associated CAA with SCD. Myocardial bridging of proximal LAD is another common anomaly and has prevalence of 0.15–25% in coronary angiograms (CAGs) [6].

Initially these anatomical variations were only published as case reports without much emphasis on its systematic study. It was pioneering efforts of Ogden and Angelini that CAA received comprehensive classifications. Ogden in 1969 [7] divided them in three basic categories as minor, major and secondary anomalies. Minor defined as variation in origin from aorta with normal distal circulation, major anomaly was either a coronary cameral fistula (CCF) or abnormal origin from PA, secondary anomaly encompassed spectrum of coronary anomalies usually associated with intracardiac defects like tetralogy, etc. Although his paper received the Young Investigators Award from American College of Cardiology for a significant step towards categorizing this once ambiguous entity, it was unable to define some critical anomalies. Angelini [8] in 1989, defined them based on statistical data and any anatomy with population prevalence of <1% was accepted as an anomaly. It was a classification by Dodge-Khatami *et al*. [9] that is also endorsed by Society of Thoracic Surgeons-Congenital Heart Surgery Committee and European Association of Thoracic Surgeons, which is in current use for its simplistic yet precise approach. It utilizes a hierarchy system and is replicated in Table 1.

**Table 1:** – Hierarchy of coronary artery classification

Hierarchy level	Anomaly
1	Coronary artery anomaly
2	Coronary anomaly: anomalous pulmonary origins of coronaries (APOC)
	Coronary anomaly: anomalous aortic origins of coronaries (AAOC)
	Coronary anomaly: congenital atresia of left main (CALM)
	Coronary anomaly: coronary arteriovenous fistula (CAVF)
	Coronary anomaly: coronary artery bridging (CB)
	Coronary anomaly: coronary artery aneurysm (CAAn)
	Coronary anomaly: coronary stenosis

### CASE SERIES

#### Case 1

**Figure 1:**
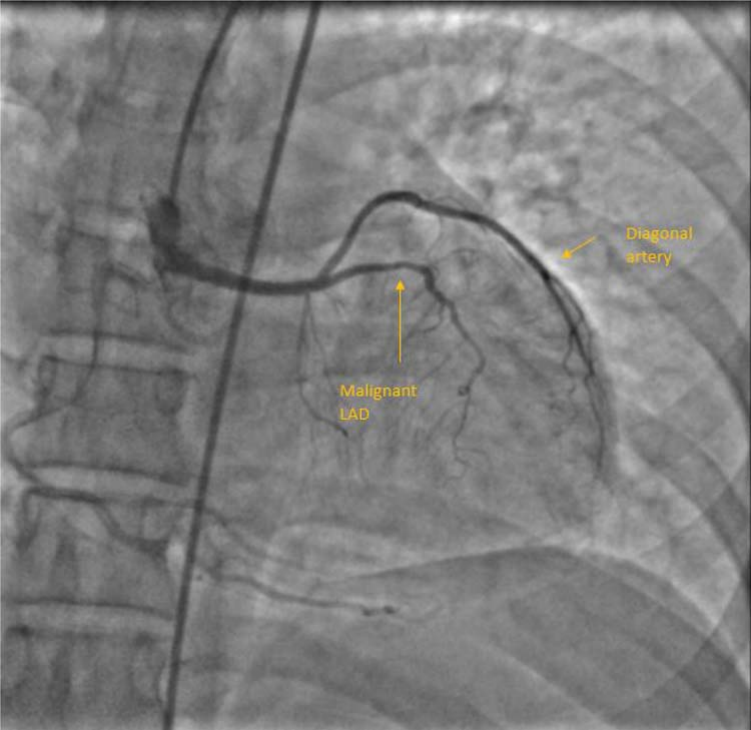
CAG with AAOC and malignant LAD course.

A 55-year-female diagnosed of having malignant LAD course 10 years back. She was fairly asymptomatic at that time and chose not to be operated. Two things changed over the next few years. She developed chest discomfort at physical activity lower than what she could previously tolerate, also developing calculous cholecystitis for which she needed to be operated. Although the motive for her to be able to undergo cholecystectomy was stronger than her exertional chest discomfort, she was well counselled and scheduled for surgery. Through median sternotomy, LIMA harvested and after cardioplegic cardiac arrest initially aortotomy done to confirm coronary origins. LAD and LCx had origination from RSOV. LAD from separate ostium, common LCx and RCA origin. Mid LAD segment was intramyocardial and bridging was released following LIMA-LAD anastomosis. Proximal LAD ligated after initial test ligation. She had an uneventful postoperative period and discharged on seventh postoperative day (POD). Figure 1 shows her CAG.

#### Case 2

**Figure 2:**
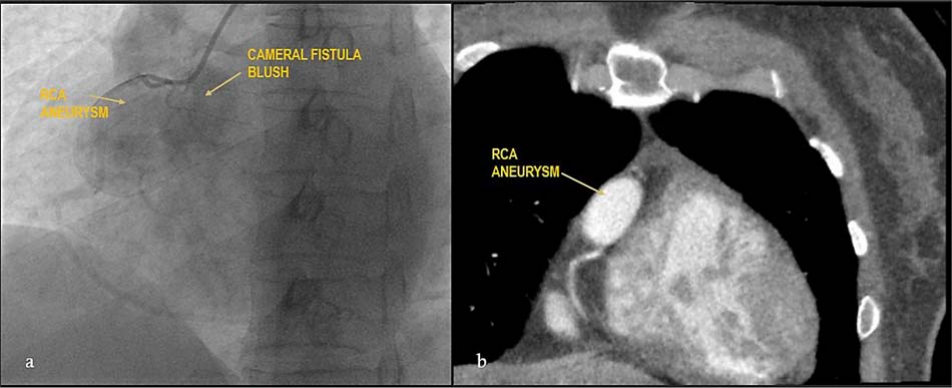
Giant RCA aneurysm with CCF. (**a**) CAG showing RCA aneurysm with RA blush; (**b**) RCA aneurysm in cardiac CT.

**Figure 3:**
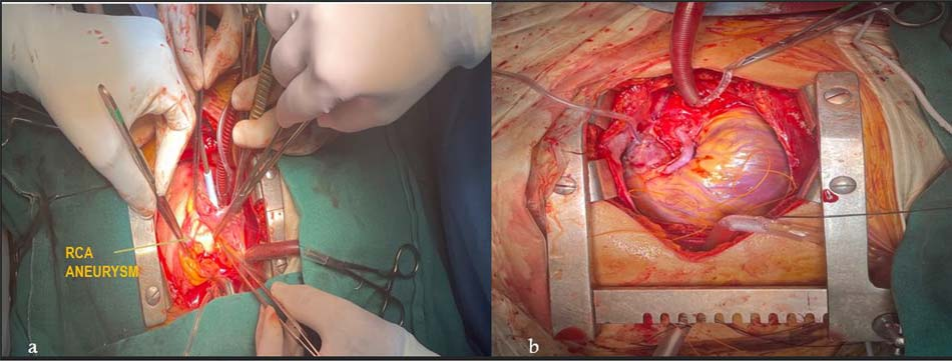
Intraoperative images. (**a**) RCA aneurysm on right angled forceps; (**b**) post RCA bypass with RSVG.

Apparently healthy 44-year-female being evaluated as a potential donor for renal transplant, her echocardiography showed dilated right atrium and ventricle with moderate tricuspid regurgitation. Gated cardiac CT was done which revealed proximal RCA aneurysm measuring 2.1×2.4 cm and CAG which revealed RCA–right atrial (RA) cameral fistula. Heart arrested with antegrade cardioplegia after aorto-bicaval cannulation. RA fistula suture ligated through right atriotomy. The aneurysm excised, RCA mobilized and reimplanted into right sinus of valsalva (RSOV). On weaning off of bypass, severe RV dysfunction observed and cardiopulmonary bypass re-initiated. RCA bypass with reverse saphenous vein graft (RSVG) was done. Figures 2 and 3 show radiological and intraoperative images. She had a stormy postoperative course with multiple episodes of ventricular arrhythmias probably attributed to sacrifice of the infundibular and nodal branch, but was eventually discharged on 11th day after surgery.

#### Case 3

**Figure 4:**
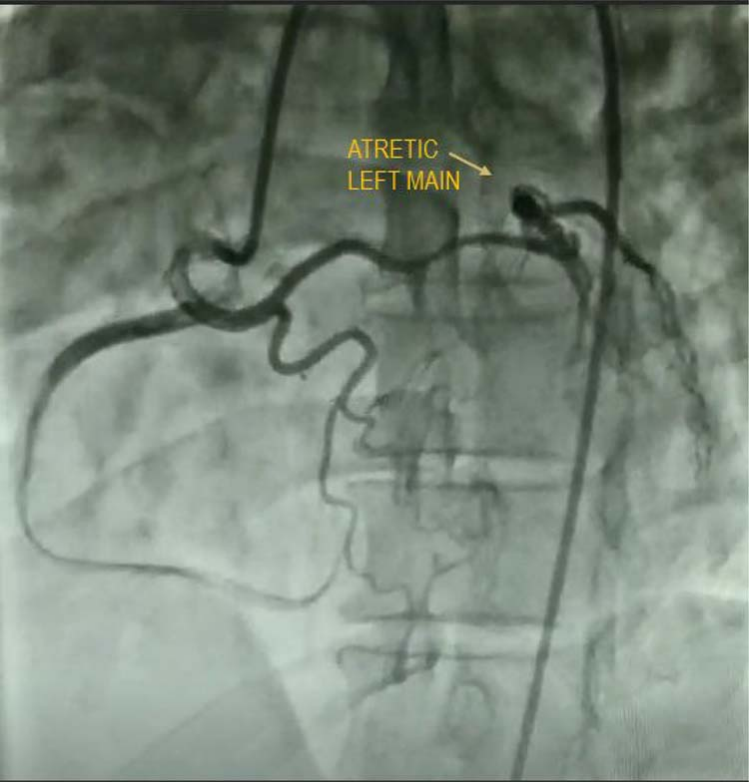
CAG showing CALM.

A quadragenarian gentleman presented to cardiology out patient department (OPD) with complaint of a central chest discomfort with normal electrocardiogram (ECG) and echocardiography. His treadmill test came positive for inducible/reversible ischemia with modified Bruce Protocol. His CAG shown in Fig. 4 reveals an atretic left main coronary artery with circulation from RCA. He was referred to us for coronary artery bypass graft (CABG) and underwent LIMA–LAD, radial artery to diagonal and obtuse marginal artery bypass with a smooth postoperative recovery.

#### Case 4

**Figure 5:**
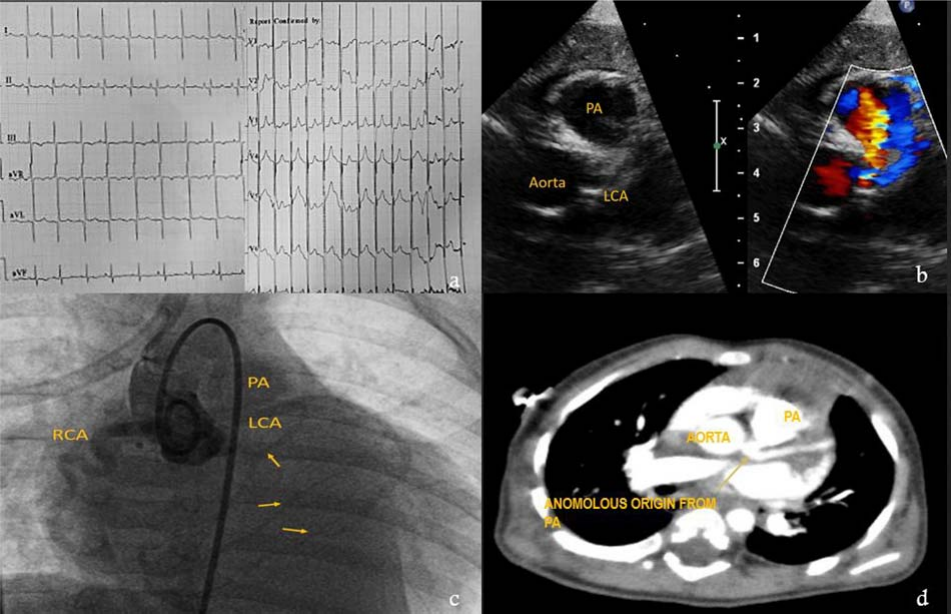
Investigations for diagnosis of ALCAPA. (**a**) ECG showing characteristic findings. (**b**) Echocardiography. (**c**) Aortic root angiogram showing coronary draining into PA. (**d**) CT angiogram.

Eight**-**month-male child admitted at a pediatric hospital for pneumonia and was referred to our pediatric cardiologist for cardiomegaly in his chest X-ray. ECG showed characteristic deep ‘q’ waves in Leads I, aVL, V5–V6 suggestive of anterolateral ischemia. Diagnosis of ALCAPA was suspected on echocardiography and confirmed on CT and root angiogram. His CT showed anomalous origin of left coronary from posterior sinus of PA and the aortic root angiogram spectacularly revealed retrograde flow from RCA to left system draining into the PA, as shown in Fig. 5. With only an ejection fraction (EF) of 20% due to coronary steal, child was scheduled for surgery at an earliest date possible following resolution of pneumonia. He underwent Modified Takeuchi repair with autologous pericardium used for creation of intrapulmonary tunnel with augmentation of PA. LV was noted to be stiff intraoperatively. First POD echo showed severe LV systolic dysfunction and severe MR. On second day, his blood pressure dropped requiring escalation of ionotropes. Veno-arterial extracorporeal membrane oxygenation (ECMO) support via central cannulation started, which improved his hemodynamics. Although successfully taken off the ECMO support on fifth POD, he developed sepsis and despite aggressive treatment with appropriate antibiotics, he developed multi organ dysfunction syndrome and succumbed on 16th POD.

## DISCUSSION

An anomalous aortic origin of coronaries independently does not manifest symptoms, but the course which the vessel takes to reach its perfusion area is clinically relevant. The course maybe prepulmonic, retroaortic, intraseptal and most importantly inter-arterial i.e. between aorta and PA. This inter-arterial course represents 5–35% risk for SCD in young individuals involved in rigorous physical activity SCD [10]. Study published by Angelini *et al*., employing intravascular ultrasound (IVUS) to assess pathophysiology of abnormal course, has provided crucial quantifiable features of anatomy such as hypoplasia, lateral compression of intramural coronary artery, possible exercise-related narrowing of the proximal ectopic segment as cause of SCD [11]. Stent placement in the proximal intramural course of vessel could be unsafe due to stent compressibility and lack of stent durability data in such circumstances [12]; hence, CABG remains the preferred approach.

CAAn has a reported incidence of 0.3–4.9%, but prevalence of giant coronary aneurysms which are defined when their diameter exceeds >4 times reference vessel or >2cm in adults and >8mm in children is as low as 0.02% [13, 14]. Atherosclerosis in adults and Kawasaki disease in children are the most frequent causes with higher prevalence among females [15]. Right system is most commonly involved (40–70%) and left main stem being rarest (3.5%) [16]. CAAn also shows simultaneous association with cameral fistula in most reported cases usually identified in young population [17]. Their approach is either by aneurysm exclusion with a covered stent or surgical. As our case had ostio-proximal giant aneurysm with cameral fistula, Heart Team Approach decided that surgery would be most appropriate.

**Figure 6:**
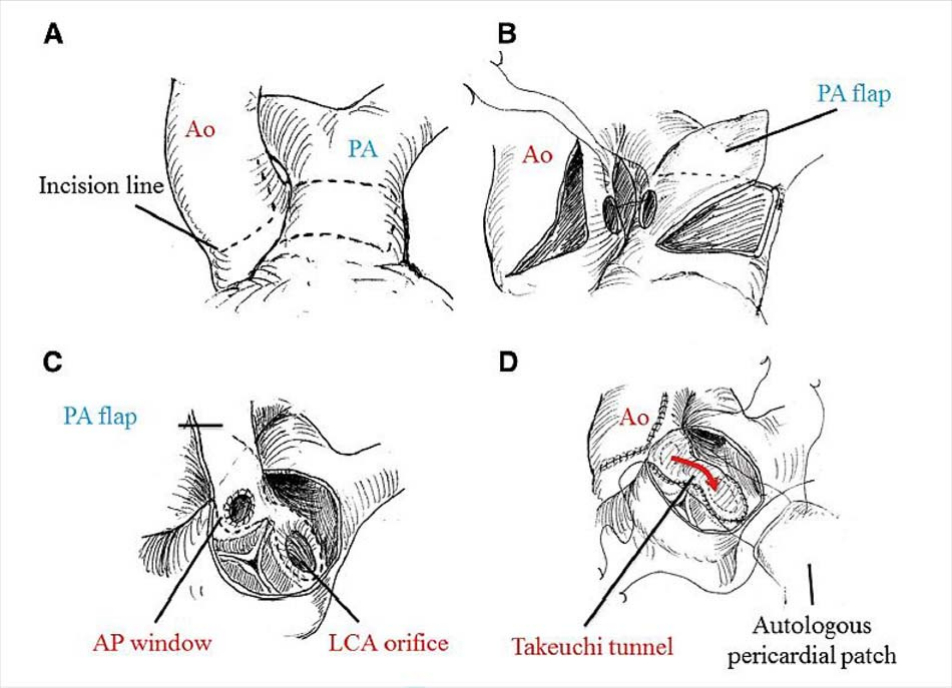
Technical details of Takeuchi technique. (**A**) aortotomy and incision of main PA (MPA). (**B**) creation of aorto-pulmonary window. (**C**) Pulmonary arterial flap suture (dashed line). (**D**) Reconstruction of MPA.  Ao, aorta; PA, pulmonary artery; AP, aortopulmonary; LCA, left coronary artery. Required permission obtained from the respective journal.

Bland *et al*. in 1933 first described clinical features with autopsy study of ALCAPA, and hence it is also named as Bland–White–Garland Syndrome [18]. Its pathophysiology principally lies in coronary steal from high pressure left coronary artery to low pressure PA[19] causing infarction of the antero-lateral free wall, congestive heart failure, ischemic mitral regurgitation. Mortality is almost 100% in untreated cases as circulation entirely depends on collaterals from RCA [20]. Ideal management is to establish dual coronary circulation which can be either by coronary translocation to aorta, bypass using subclavian or internal mammary artery, Modified Takeuchi procedure which creates and intrapulmonary tunnel and of historical interest proximal ligation of left system [21]. Takeuchi procedure is the procedure of choice when the LCA is far from aorta usually in non-facing sinus, and the technical details are depicted in Fig. 6. Lange *et al*. [22] in their study reported 30-day mortality of 14.3% and EF of <35% as independent predictor of mortality, the child in this study had an EF of 20%.

CAA generally remains asymptomatic in adults but may have fatal presentation in certain variants like heart failure and infarction in ALCAPA, SCD when coronary artery has an inter-arterial course. Their management can be limited to conservative management, stent placement to surgical procedures like CABG, creation of a dual coronary system and a highly variable postoperative course.
